# Size–strain separation in diffraction line profile analysis

**DOI:** 10.1107/S1600576718005411

**Published:** 2018-05-29

**Authors:** P. Scardi, M. Ermrich, A. Fitch, E-Wen Huang, R. Jardin, R. Kuzel, A. Leineweber, A. Mendoza Cuevas, S. T. Misture, L. Rebuffi, Christian Schimpf

**Affiliations:** a Department of Civil, Environmental and Mechanical Engineering, University of Trento, Trento, Italy; b Röntgenlabor Dr Ermrich, Am Kandelborn 7, D-64354 Reinheim, Germany; c ESRF, 71 avenue des Martyrs, CS 40220, 38043 Grenoble Cedex, France; d Department of Materials Science and Engineering, National Chiao Tung University, Hsinchu, Taiwan; e Bruker AXS GmbH, Oestliche Rheinbrueckenstrasse 49, 76187 Karlsruhe, Germany; f Department of Condensed Matter Physics, Charles University, Prague, Czech Republic; g Institute of Materials Science, TU Bergakademie Freiberg, D-09599 Freiberg, Germany; h Archaeometry Laboratory, Havana’s Historian Office, University San Geronimo de La Habana, Habana, Vieja, Cuba; i Multidisciplinary Laboratory, ICTP, I-34151 Trieste, Italy; j Alfred University, Alfred, NY 14802, USA; k Elettra-Sincrotrone Trieste, Trieste, Italy

**Keywords:** line profile analysis, powder diffraction, microstrain, crystalline domain size

## Abstract

Separation of size and strain effects on diffraction line profiles has been studied in a round robin involving laboratory instruments and synchrotron radiation beamlines operating with different radiation, optics, detectors and experimental configurations. The studied sample, an extensively ball milled iron alloy powder, provides an ideal test case, as domain size broadening and strain broadening are of comparable size. The high energy available at some synchrotron radiation beamlines provides the best conditions for an accurate analysis of the line profiles, as the size–strain separation clearly benefits from a large number of Bragg peaks in the pattern; high counts, reliable intensity values in low-absorption conditions, smooth background and data collection at different temperatures also support the possibility to include diffuse scattering in the analysis, for the most reliable assessment of the line broadening effect. However, results of the round robin show that good quality information on domain size distribution and microstrain can also be obtained using standard laboratory equipment, even when patterns include relatively few Bragg peaks, provided that the data are of good quality in terms of high counts and low and smooth background.

## Abstract 2

Separation of size and strain effects on diffraction line profiles has been studied in a round robin involving laboratory instruments and synchrotron radiation beamlines operating with different radiation, optics, detectors and experimental configurations. The studied sample, an extensively ball milled iron alloy powder, provides an ideal test case, as domain size broadening and strain broadening are of comparable size.

## 1. Introduction   

X-ray diffraction (XRD) line profile analysis (LPA) is frequently used to gain insight into the microstructure of crystalline materials, often complementing evidence provided by electron microscopy. The most commonly sought information concerns the size of the crystalline domains, related to the inverse of the peak width by the well known Scherrer equation (Scherrer, 1918 ▸). A large variety of LPA methods have been proposed, to account for the instrumental contribution to the line profile and for specific features of the microstructure, like line defects, faulting or anti-phase boundaries, to mention just a few. Even though LPA methods are still the object of active research, well established procedures can be found in several textbooks (Klug & Alexander, 1974 ▸; Warren, 1990 ▸; Guinebretière, 2007 ▸), technical monographs and special issues (Snyder *et al.*, 1999 ▸; Mittemeijer & Scardi, 2004 ▸; Dinnebier & Billinge, 2008 ▸; Scardi & Dinnebier, 2010 ▸; Mittemeijer & Welzel, 2013 ▸).

In spite of the popularity of LPA, few studies have focused on the reliability of the results, and still no specific reference material is available to test measurement procedures and LPA, unless peak broadening is solely due to the finite size of the crystalline domains. In a round robin organized about 15 years ago on nanocrystalline ceria (Balzar *et al.*, 2004 ▸), XRD peak broadening was found to be mostly related to the small size of the crystalline domains, with just minor contributions from the instrument and from a microstrain of unspecific origin. Similarly, after extensive research work (Armstrong *et al.*, 2005 ▸; Cline *et al.*, 2013 ▸), NIST recently released a new Standard Reference Material, SRM 1979 (zinc oxide), which is intended as a standard for crystallite size analysis, with just minor contributions from faulting and apparently no strain effects (Cline *et al.*, 2016 ▸).

Size-induced peak broadening is well known, both in terms of theory (see *e.g.* Stokes & Wilson, 1942 ▸; Bertaut, 1950 ▸; Wilson, 1962 ▸; Langford & Wilson, 1978 ▸; Scardi & Leoni, 2001 ▸) and experimentally, but most often peak broadening has several sources. A common case concerns the effects of atomic displacements, as invariably found in plastically deformed materials with the presence of line defects (Wilson, 1952 ▸, 1955 ▸; Krivoglaz & Ryaboshapka, 1963 ▸; Wilkens, 1970*a* ▸,*b* ▸), but also and more generally in polycrystalline aggregates, with the presence of grain boundaries (Rebuffi *et al.*, 2016 ▸), and in nanocrystalline powders, with the presence of surface relaxation (Scardi *et al.*, 2015 ▸). In these common cases the primary task of LPA is to separate the effects of finite domain size, including the size dispersion invariably present in real samples, from the broadening caused by atomic displacement (also known as microstrain broadening).

It is, therefore, of interest (i) to compare data collection strategies for specimens with domain size and microstrain effects, (ii) to assess the reliability of results and the effectiveness of procedures to separate instrumental contribution, size and microstrain effects, and (iii) to identify potential candidate materials for new standards for LPA. The present work is mostly focused on (i), to assess the real possibility of separating size and strain effects and how the result is influenced by the quality and quantity of information available from experiments performed with different optics and X-ray wavelengths, using both commercially available equipment and synchrotron radiation (SR) XRD instruments. To this end we studied a ball milled metal powder which we consider as a nearly ideal specimen, as it is stable, is available in large amounts from the same production batch, and shows comparable broadening effects of finite domain size and strain caused by plastic deformation. The Fe–1.5 wt% Mo powder used in this work also has the advantage of having been characterized extensively in earlier work (Rebuffi *et al.*, 2016 ▸; Scardi *et al.*, 2017 ▸), which makes clear which effects contribute to the line broadening. There is no intention in this study to propose the ‘best’ method to perform a separation of size and strain effects, but a well documented state-of-the-art procedure is used with the purpose of showing differences between the different experimental setups and data quality, and to demonstrate, in general, the possibility of separating size and strain contributions. All experimental data, collected by several laboratories participating in this project, are made available to the community of XRD users, for future reference and to test methods and procedures. This work might also seed the development of new SRMs for the separation of size and strain contributions to line broadening.

## 2. Experimental   

### 2.1. Ball milled iron alloy sample   

The studied sample is a commercial powder of iron alloyed with 1.5 wt% Mo (Astaloy Mo, Höganäs) extensively ground (64 h) in a planetary ball mill (Fritsch P4). The pristine powder is composed of the ferritic phase [body centred cubic α-iron, space group 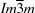 (229), unit-cell parameter 2.870 Å]. Details of the preparation, chemical analysis, electron microscopy and LPA can be found in the cited literature (Rebuffi *et al.*, 2016 ▸; Scardi *et al.*, 2017 ▸).

Powder samples from the same batch [batch 4a; see Rebuffi (2015 ▸) for details] were distributed to each participating laboratory and measured under the different experimental conditions provided by the specific instrumentation (see §2.2). All samples are therefore equivalent, as shown in previous work (Rebuffi *et al.*, 2016 ▸), which also confirmed that the sample is stable over time, for more than ten years (Troian *et al.*, 2015 ▸).

### 2.2. Diffraction measurements: instruments and setups   

This study concerns how data quantity and quality influence results in terms of accuracy and reliability. Thus we collected ‘top quality/quantity’ data exploiting SR beamline instruments operated with high (≥15 keV) energy, but also a number of patterns from traditional laboratory instruments, including some data sets of apparently ‘poor’ statistical quality (*e.g.* collected in a short time) and/or encompassing few Bragg peaks. This was done intentionally, to highlight the diversity of the data sets that are regularly collected in measurement laboratories, and also to show how data quantity/quality influence the results, for example the different numbers of peaks in measured patterns, counting statistics, instrumental resolution, the intrinsic quality of the peak profile modelling, radiation energy *etc*.

**Figure 1:**
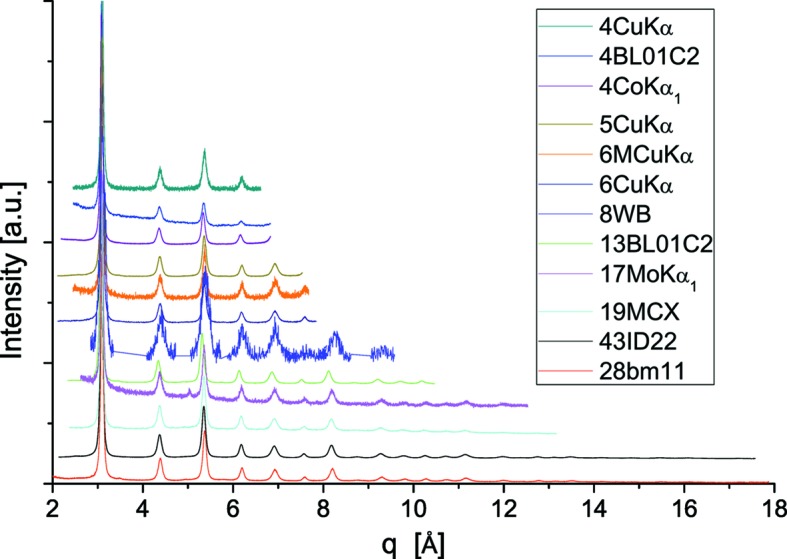
Synoptic view of all data collected for this study. All patterns were collected at room temperature [with the exception of 11bm, where data (not shown here) are also available at 100 and 200 K]. To allow for a direct comparison, all patterns are shown as intensity in arbitrary units *versus q*, and are scaled upward according to the extension in *q* space.

The instruments and experimental conditions used in this study are listed in Table 1 ▸, with the corresponding diffraction patterns shown in Fig. 1 ▸. For an easier visual comparison, intensities are reported as a function of *q* = 4πsinθ/λ (where θ is half the scattering angle and λ is the wavelength of the incident radiation) and appropriately rescaled.

## 3. Results and discussion   

### 3.1. XRD pattern modelling procedure   

#### 3.1.1. Instrumental profile   

The instrumental profile (IP) was experimentally evaluated from the powder pattern of standard materials (see Table 1 ▸), with a whole pattern fitting procedure. The diffraction peak profiles of each spectral component (*e.g.* *K*α_1_ and *K*α_2_) were empirically modelled by pseudo-Voigt functions, defined as
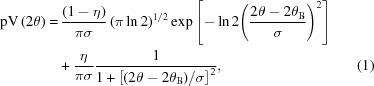
where the adjustable parameters are the Lorentzian fraction η, the peak position 2θ_B_, the full width at half-maximum (FWHM = 2σ) and a global scaling factor, not shown in equation (1). When data sets provide an abscissa different from 2θ, *e.g.* *s* = 2sinθ/λ or *q* = 2π*s*, equation (1) is modified accordingly. A Chebyshev polynomial was used to account for the background in the powder patterns.

The IP was parameterized according to the Caglioti formula (Caglioti *et al.*, 1958 ▸) for FWHM, while η follows a polynomial in θ:
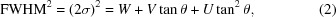


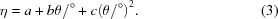
The parameters of equations (2) (*U*, *V*, *W*) and (3) (*a*, *b*, *c*) were refined for each instrumental setup so that afterwards the IP was known at any desired diffraction angle. Aberrations on peak position, as shown by Wilson (1963 ▸), can be modelled as a primary effect on peak centroid, which is shifted by
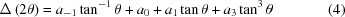
with coefficients *a*_−1_, *a*_0_, *a*_1_, *a*_3_ also refined from the experimental pattern of the standard. A second-order coefficient (*a*_2_) might also be added, although theory (Wilson, 1963 ▸) does not predict its existence for powder diffractometers.

**Figure 2:**
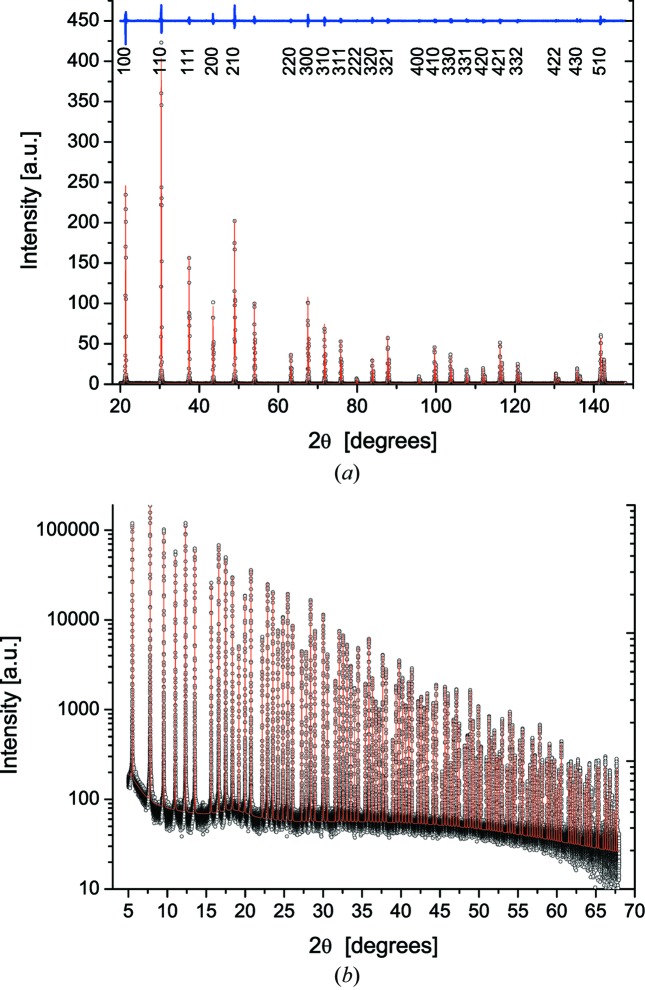
Whole pattern fitting of LaB_6_ standard data, using pV profile functions. (*a*) Cu *K*α_1,2_ data (6CuKα) with indication of residual (above, difference between experimental and fitting profile) and Miller indices. (*b*) 43ID22 with 31 keV radiation: and modelling using pV profile functions for 119 peaks of LaB_6_ (NIST SRM660b). Note the linear scale in (*a*) *versus* the logarithmic scale in (*b*). See supporting information for results for all instruments in this study.

**Figure 3:**
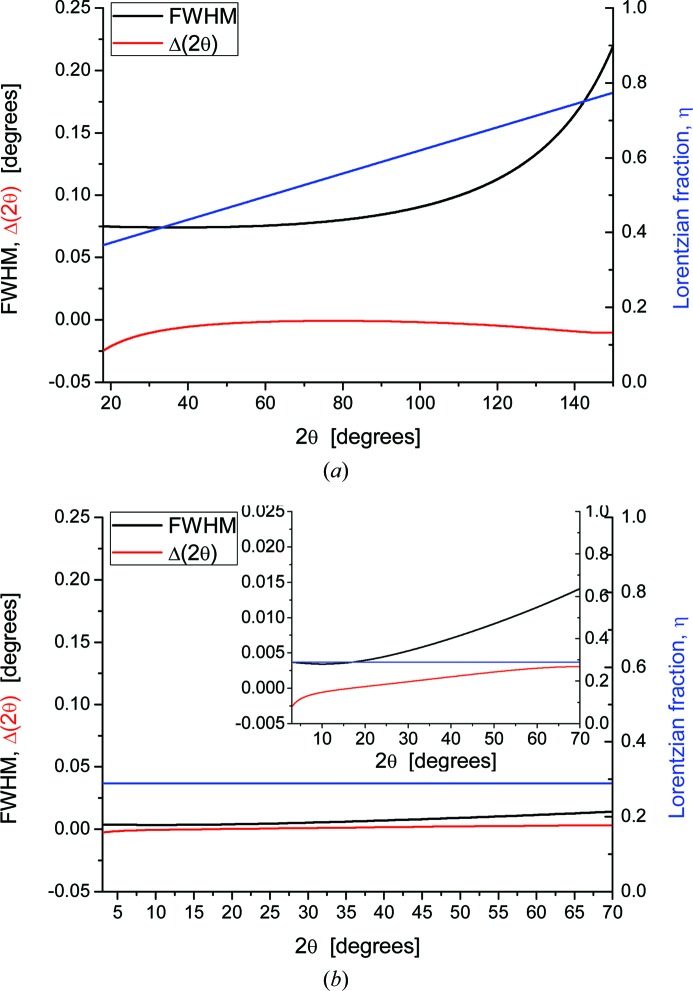
(*a*) Parameterization of IP (FWHM and η) from the data in Fig. 2 ▸(*a*). Refined coefficients for the 6CuKα laboratory instrument (Fig. 2 ▸ *a*) are *W* = 5.906369 × 10^−3^, *V* = −2.502974 × 10^−3^, *U* = 3.712228 × 10^−3^, *a* = 3.107787 × 10^−1^, *b* = 6.175673 × 10^−3^, *c* = 0. The lower line is the tanθ polynomial correcting aberrations on peak position [equation (4)] (Wilson, 1963 ▸), with refined parameters *a*_−1_ = −5.978103 × 10^−4^, *a*_0_ = 1.437456 × 10^−2^, *a*_1_ = −9.797414 × 10^−3^, *a*_2_ = 0, *a*_3_ = 2.635631 × 10^−4^. (*b*) The same analysis is made for the pattern in Fig. 2 ▸(*b*), 43ID22 with 31 keV radiation, showing a much narrower IP than in (*a*), and nearly negligible peak position correction; the inset shows data with a ×10 expansion of left ordinate axis. For most instruments η varies linearly with θ, as in the cases in this figure, or is even constant as for SR data. See supporting information for results for all instruments in this study.

As an example of two typical configurations of this study, a laboratory instrument and a synchrotron radiation beamline, Fig. 2 ▸ shows experimental and modelled powder patterns of LaB_6_, NIST SRM660b [laboratory (*a*) and SR (*b*)], and corresponding results of the parameterizations are given in Fig. 3 ▸: IP, equations (2) and (3), and peak position aberrations, equation (4). Analogous results for all instruments in this study are reported in the supporting information.

The results of the parameterization of equations (2) and (3) can readily be used to compute the Fourier transform (FT) of the IP component, to be used in the modelling of the studied sample in a convolution with the other effects contributing to the line profile (see below). To this purpose it is convenient to introduce
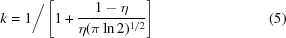
such that the FT of the pV peak function representing the IP can be written as
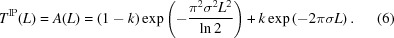
Here, *L* is the Fourier variable, with dimension of length. The FT has only a real component because, to a reasonable approximation, the IP peaks in this study are symmetrical in the 2θ range of interest. Better and more sophisticated descriptions of the IP are of course possible, for example by the fundamental parameter approach (Cheary & Coelho, 1992 ▸). This may be necessary if the IP accounts for a significant part of the total experimental profile; in the present case, the most relevant line broadening effect is caused by the microstructure, so that details of the IP are of lesser importance.

#### 3.1.2. Whole powder pattern modelling   

Once the parametric expressions for FWHM and η are known, the FT of equation (6) can be used to convolve the IP with profile components arising from the microstructure of the ball milled powder. As shown by previous studies on this powder (Rebuffi *et al.*, 2016 ▸; Scardi *et al.*, 2017 ▸), major contributions to the line profile are given by the finite domain size and by the inhomogeneous strain, a frequent condition for ball milled materials. Following a whole powder pattern modelling (WPPM) approach (Scardi, 2008 ▸) based on the Fourier theorem for convolutions, peak profiles can be modelled as
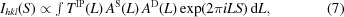
where 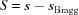, while the proportionality symbol includes known constants and trigonometric functions, as well as structural information (square modulus of the structure factor) and the thermal (Debye–Waller) factor (Warren, 1990 ▸).

The line profile components of the microstructure can be conveniently modelled by considering the following:

(i) A size broadening effect, 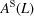, from spherical domains with a lognormal distribution of diameters, 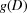 (Scardi, 2008 ▸),
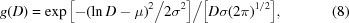


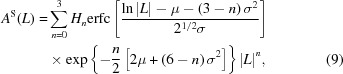
where μ and σ^2^ are, respectively, the lognormal mean and variance, and *H_n_* are numerical coefficients for the domain shape (for spherical domains, *H*_0_ = 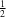, *H*_1_ = −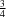, *H*_2_ = 0, *H*_3_ = 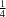); the distribution mean is 〈*D*〉 = exp(μ + 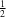
σ^2^), the standard deviation is s.d. = {[exp(σ^2^) − 1]exp(2μ + σ^2^)}^1/2^. Transmission electron microscopy (TEM) studies reported previously (Rebuffi *et al.*, 2016 ▸; Scardi *et al.*, 2017 ▸) have shown that the crystalline domains after extensive ball milling are equiaxed on average, so that the assumption of spherical domain shape seems appropriate.

(ii) A strain broadening, 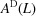,
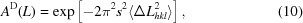
where 〈Δ*L* ^2^ *_hkl_*〉, the variance of the displacement distribution for pairs of atoms at distance *L* along [*hkl*], is a function of *L* and can be described by the Popa–Adler–Houska (PAH) model (Adler & Houska, 1979 ▸; Popa, 1998 ▸; Scardi *et al.*, 2015 ▸) or by the Wilkens model (Wilkens, 1970*a* ▸,*b* ▸; Scardi, 2008 ▸, and references therein) for dislocations:
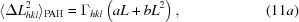


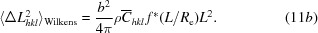
Here, *a* and *b* are free (refinable) coefficients in equation (11*a*) (Scardi *et al.*, 2015 ▸; Leonardi & Scardi, 2016 ▸); *b* ^2^ in equation (11*b*) is the square modulus of the Burgers vector, ρ is the average dislocation density, *R*_e_ is the effective outer cut-off radius of the dislocation system and f* is the so-called Wilkens function (Wilkens, 1970*a* ▸,*b* ▸). Both strain models account for broadening anisotropy according to a fourth-order invariant form of the Miller indices (fourth-order polynomial in *hkl*) (Scardi, 2008 ▸; Martinez-Garcia *et al.*, 2009 ▸; Ungár *et al.*, 1999 ▸):
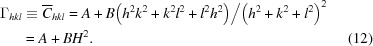

*Γ_hkl_* and 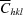 are functionally equivalent, with the important difference that in the former case *A* and *B* are just additional free parameters, to be adjusted according to the specific peak broadening anisotropy in the studied sample, whereas for 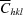 (average contrast factor), *A* and *B* can be calculated from the elastic constants and dislocation slip system (Martinez-Garcia *et al.*, 2009 ▸).

The WPPM procedure can also account for the temperature diffuse scattering (TDS), according to Warren’s model (Warren, 1990 ▸) extended to nanocrystalline domains (Beyerlein *et al.*, 2012 ▸). Among all available data sets, only 28bm11 was collected at three temperatures (100, 200 and 300 K), thus allowing for a detailed analysis of the static and dynamic contributions to the diffuse scattering (Scardi *et al.*, 2017 ▸). However, TDS was also included in the analysis of 43ID22, as the room-temperature data were collected with a high-energy (31 keV)/high-brilliance beam, providing the longest list of Bragg peaks of the present study, under controlled (*i.e.* negligible) absorption conditions, which make possible a reliable inclusion of the thermal scattering effects. As pointed out in the cited paper, this ball milled sample is composed of a ferritic (body centred cubic) iron phase, but contamination from the milling vials (composed of NiCr steel) tends to stabilize a small fraction of austenite, also included in the WPPM. This secondary phase can only be identified in some of the SR powder patterns.

### 3.2. Size–strain separation   

**Figure 4:**
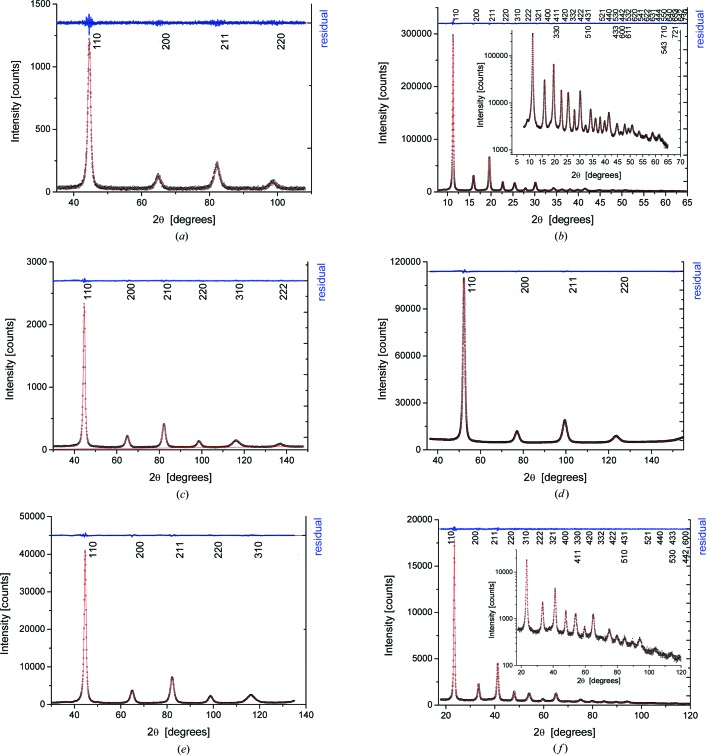

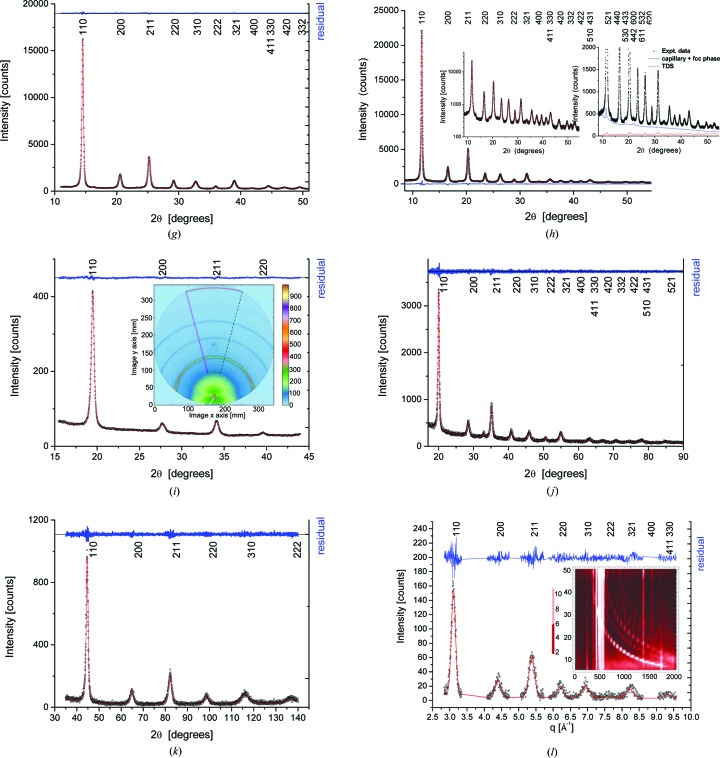
Experimental data (circular data points), modelling (line) and their difference (residual, upper line). (*a*) 4CuKα; (*b*) 43ID22, with detail in log scale in the inset; (*c*) 6CuKα; (*d*) 4CoKα_1_; (*e*) 5CuKα; (*f*) 19MCX 15 keV; (*g*) 13BL01C2 24 keV; (*h*) 28bm11 30 keV; (*i*) 4BL01C2 18 keV, with detail of the imaging plate reading in the inset; (*j*) 17MoKα_1_ (the unindexed peak at 33° is a spurious effect of scattering from the glass capillary); (*k*) 6MCuKα; (*l*) 8WB, with the density plot of angle *versus* energy shown in the inset. Miller indices are shown in each plot.

All data sets can be modelled adequately by the WPPM procedure described above. This is true of patterns with just four peaks of the ferritic iron phase and relatively low quality statistics like in Fig. 4 ▸(*a*) (4CuKα), as well as for top-quality data, extended to large *q*, as in Fig. 4 ▸(*b*), showing the experimental pattern and modelling of the 43ID22 pattern. Results for all other data sets are reported in Figs. 4 ▸(*c*)–4 ▸(*l*).

**Figure 5:**
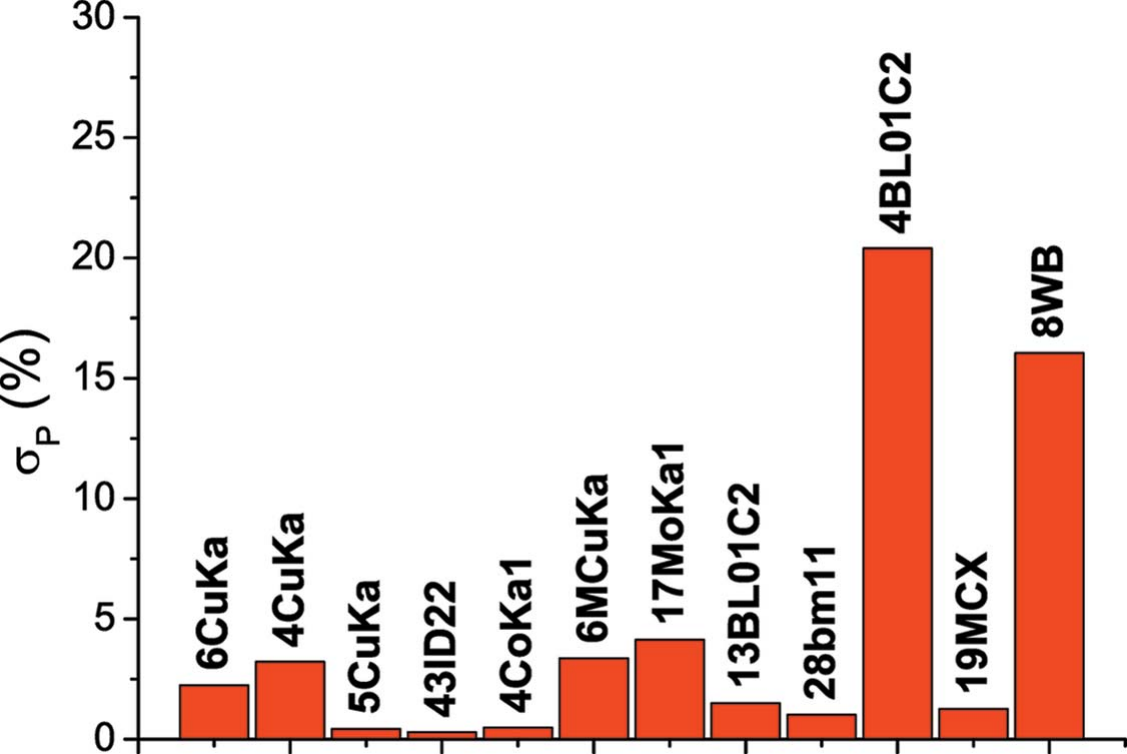
Comparison of counting statistics of the different data sets, expressed as standard deviation of the intensity distribution (Klug & Alexander, 1974 ▸), 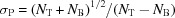, where *N*_T_ and *N*_B_ are, respectively, total and background intensity.

As a complement to the WPPM of Fig. 4 ▸, it is useful to compare the statistical quality of the different data sets. If *N*_T_ is the total intensity, including diffracted signal and background, and *N*_B_ is the background intensity from the WPPM, the standard deviation of the intensity distribution can be estimated as 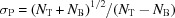 (Klug & Alexander, 1974 ▸). As shown in Fig. 5 ▸, SR data give 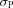 < 2%, with the exception of 4BL01C2, which was measured in a short time (30 s) and with a high background from the two-dimensional detector. The laboratory measurements are above this threshold, with the exception of 4CoKα_1_ and 5CuKα, where the high count rate and smooth background give a low standard deviation. The values of 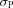 are also quite high for 8WB, which is a noisy data set: the consequences for the size–strain analysis are discussed in the following.

## 4. WPPM results   

**Figure 6:**
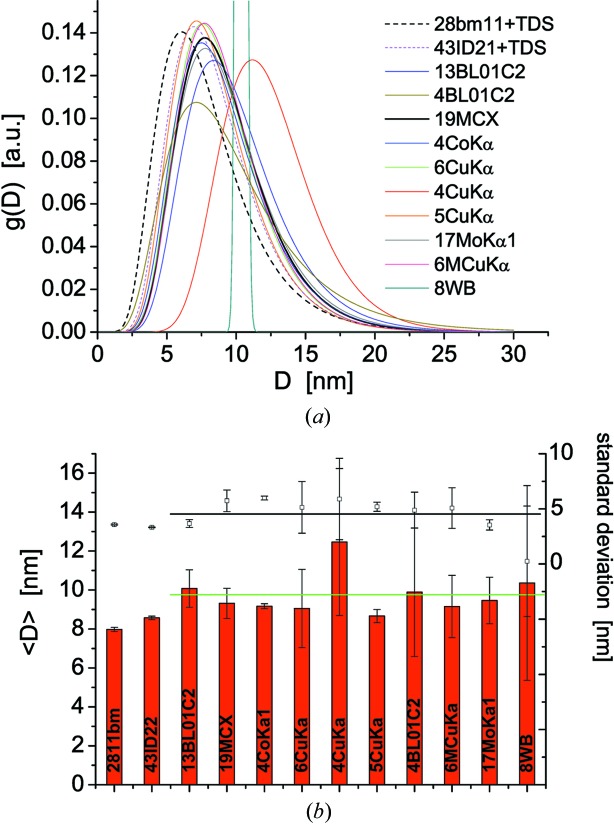
(*a*) Lognormal size distribution, *g*(*D*), from WPPM of all patterns in this study; (*b*) mean size, 〈*D*〉, and standard deviation of the distribution. The green line in (*b*) is the weighted average of all mean sizes (except 28bm11 and 43ID22, where WPPM included the TDS contribution: see text for details).

Fig. 6 ▸ shows the size distribution obtained by WPPM for all patterns in this study. Most curves cluster around the expected result, confirmed by previous analysis and electron microscopy, here reproduced as the black dashed curve: the results for 28bm11 were obtained using equation (7), including the TDS contribution, with a simultaneous analysis of three data sets collected at 100, 200 and 300 K, respectively (Scardi *et al.*, 2017 ▸). Laboratory instruments tend to overestimate slightly the extracted value of average crystallite size, partly because the TDS is not included, but especially as a result of the lower number of peaks in the patterns. Also SR beamlines with energy lower than the 30–31 keV of 28bm11 and 43ID22 fall into this category, but the results, globally speaking, are in good agreement.

Small s.u. values for mean domain size and standard deviation of the domain size distribution (Fig. 6 ▸ *b*) are obtained from data sets with low 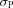. Whether it is achieved by the high brilliance of the sources and/or long counting times, data quality directly affects the reliability of the results. The results from the 4CuKα and 8WB data sets lie outside the group: the first has a ∼30% overestimated 〈*D*〉, while the second gives unreliable results for the standard deviation of the distribution and an overestimated mean size. These two data sets combine two main limitations: (i) few peak profiles in the pattern and (ii) poor statistics, in terms of noisy data and high background. Also the 4BL01C2 data (from an image plate detector), with just four peaks in the pattern collected in a short time, exhibit relatively poor statistics and consequently provide a reasonable mean size but a large s.u. (Fig. 6 ▸ *b*).

Strain broadening can be modelled equally well by equations (11*a*) and (11*b*), with no significant differences in the size distributions just discussed. Although the Wilkens model might appear more appealing than PAH, as the former is based on a specific physical model for dislocation line broadening, it is also true that the observed strain broadening, as for most plastically deformed materials, is not solely due to dislocations. Indeed, previous work on the studied sample has shown that if strain broadening is attributed to dislocations only, even assuming a cut-off radius as large as the domain size (*R*_e_ ≃ 〈*D*〉 = 8.2 nm), the calculated dislocation density is quite high, ρ = 3.2 (4) × 10^16^ m^−2^. This value corresponds to an average of nearly two dislocations per nanocrystalline domain, a condition clearly proven wrong by electron microscopy observations (Rebuffi *et al.*, 2016 ▸) and by molecular dynamics (MD) simulations (Scardi *et al.*, 2017 ▸; Leonardi & Scardi, 2016 ▸). Dislocation dipoles have been found in a few domains of the ball milled powder observed by high-resolution TEM, but apparently most nanocrystals do not contain any dislocations. As shown by MD simulations, much of the inhomogeneous strain contributing to the line broadening originates from the grain boundary region, where dislocations tend to be absorbed after slipping across grains; in this process the strain field of the dislocation may decrease but it does not disappear, and inhomogeneous strain tends to build up during the high-energy milling process (Rebuffi *et al.*, 2016 ▸).

Therefore, to avoid obtaining a dislocation density which is clearly overestimated, we assume that the strain broadening in this sample is given by a combination of grain boundary effects with dislocations in only a few domains. The strain broadening is then more adequately described phenomenologically by the best fit values of *A*, *B*, *a* and *b* in equations (11) and (12). This representation of the standard deviation of the displacement distribution, 〈Δ*L* ^2^〉^1/2^, as a function of the distance *L* along given crystallographic directions [*hkl*] in the crystalline domain was introduced by B. E. Warren in one of the early papers on his well known Fourier method (Warren & Averbach, 1950 ▸; Warren, 1990 ▸). Most importantly, the Warren plots do not require commitment to a specific model to justify the observed strain broadening, which is appropriate to the purpose of this work, *i.e.* to compare results obtained from different instruments, geometries and data collection conditions. Note that analogous results can be reported as a ‘microstrain’ plot (*i.e.* 〈*∊* ^2^〉^1/2^ = 〈Δ*L* ^2^〉^1/2^/*L versus L*), easily derived from the Warren plot.

**Figure 7:**
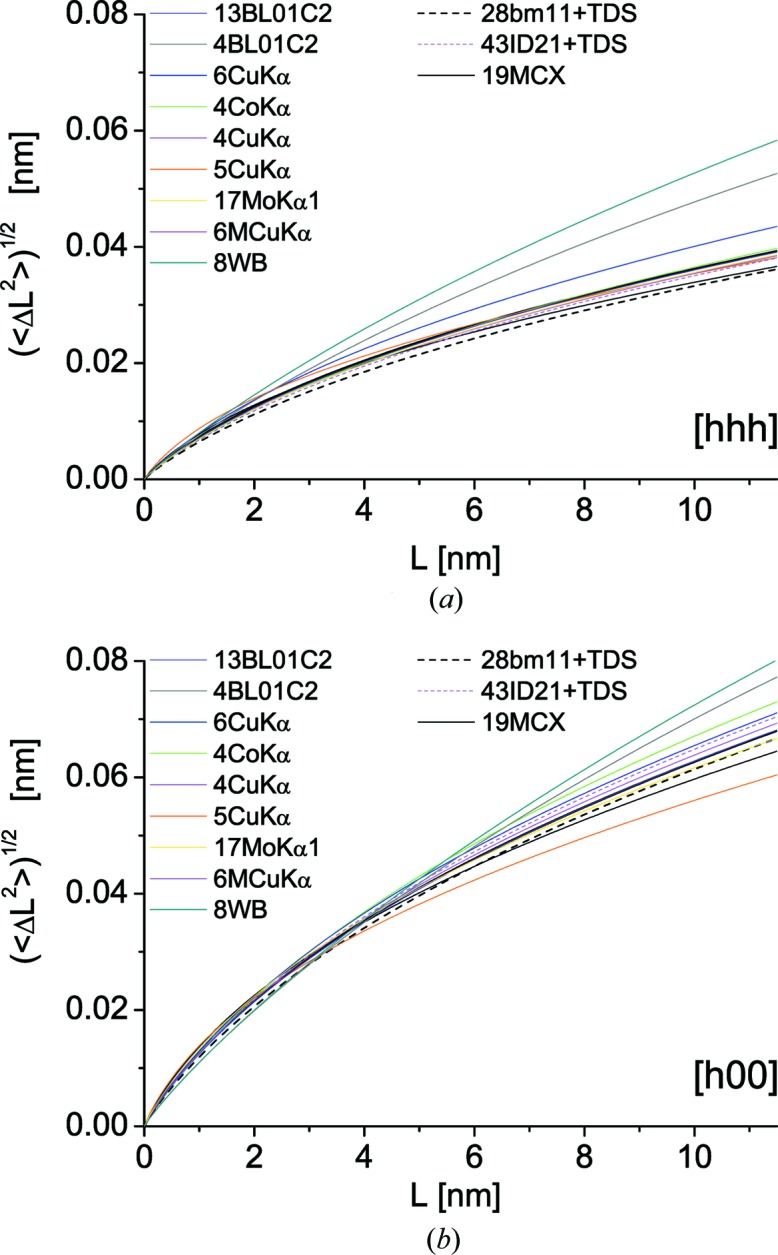
Warren plots for all samples in the study. Trend of the variance of the atomic displacement distribution as a function of the distance *L* between pairs of atoms along the two directions, (*a*) [*hhh*] and (*b*) [*h*00], corresponding to soft and stiff elastic response in ferritic iron, respectively.

Fig. 7 ▸ shows Warren plots for two crystallographic directions, [*hhh*] (*a*) and [*h*00] (*b*), corresponding, respectively, to the stiffest and softest crystallographic directions in cubic metals like ferritic iron. As expected, the 〈Δ*L* ^2^〉^1/2^ values are higher along [*h*00] than [*hhh*], thus representing the corresponding upper and lower bounds of the broadening effect. As with the size broadening component, the results from most data sets tend to group about those of SR beamlines (11bm, ID22 and MCX), apart from 8WB and 4BL01C2 which overestimate the 〈Δ*L* ^2^〉^1/2^ trends. As already noted, these data sets comprise too few peaks for a reliable separation of size/strain effects, especially for the concurrently poor counting statistics/high background leading to a high 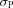.

Apart from these data sets, which demonstrate the limitations of collecting data over *q* ranges that are too narrow and the well known effects of poor counting statistics, the overall result of WPPM in this study is remarkably good. The size and strain values are reasonably close to the expected results. It is of course ideal to collect data by SR XRD up to high *q*, encompassing tens of peak profiles with good statistics (low noise, low background), with high instrumental resolution (*i.e.* narrow IP) and at different temperatures for a reliable assessment of the TDS contribution; but the present results clearly demonstrate that an effective and even reliable size–strain separation can be achieved with much less information, on easily accessible laboratory instruments equipped with conventional sources, and even with the few peaks observable by relatively low energy X-rays (*e.g.* 4CoKα_1_ data), provided that the measurement statistics are good.

The overall agreement amongst measurement configurations found in this study is partly related to the nature of the studied powder sample, where size and strain broadening effects are easily visible and of comparable magnitude. Samples with little peak broadening, *i.e.* a microstructural broadening that is small with respect to the IP component, and samples with a marked predominance of one of the two effects (size or strain) might give less positive results when comparing different instruments and data collection conditions.

## 5. Integral breadth analysis   

Integral breadth (IB) methods (Klug & Alexander, 1974 ▸) are simple and informative, although they present some issues in their practical use and the reliability of the results [see Scardi *et al.* (2004 ▸) for a critical review]. The most used method is based on the so-called Williamson–Hall (WH) plot (Williamson & Hall, 1953 ▸), with modifications to include effects of faulting and anisotropy [so-called modified WH (mWH) (Ungár *et al.*, 1999 ▸)].

Determination of IB values is not so straightforward. Problems arise from (i) the instrumental profile contribution, which must be correctly removed, and (ii) signal overlap (between peaks and with background), which must also be considered. WPPM can address (i) and (ii), as IBs can be obtained numerically by exploiting the properties of Fourier transforms:
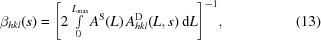
where the integral extends to *L*_max_, the maximum distance along [*hkl*] in the crystalline domain. Equation (13) intrinsically solves problems with IP contributions and overlapping, and provides IBs, 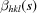, which are as good as the profile modelling (see results shown previously).

**Figure 8:**
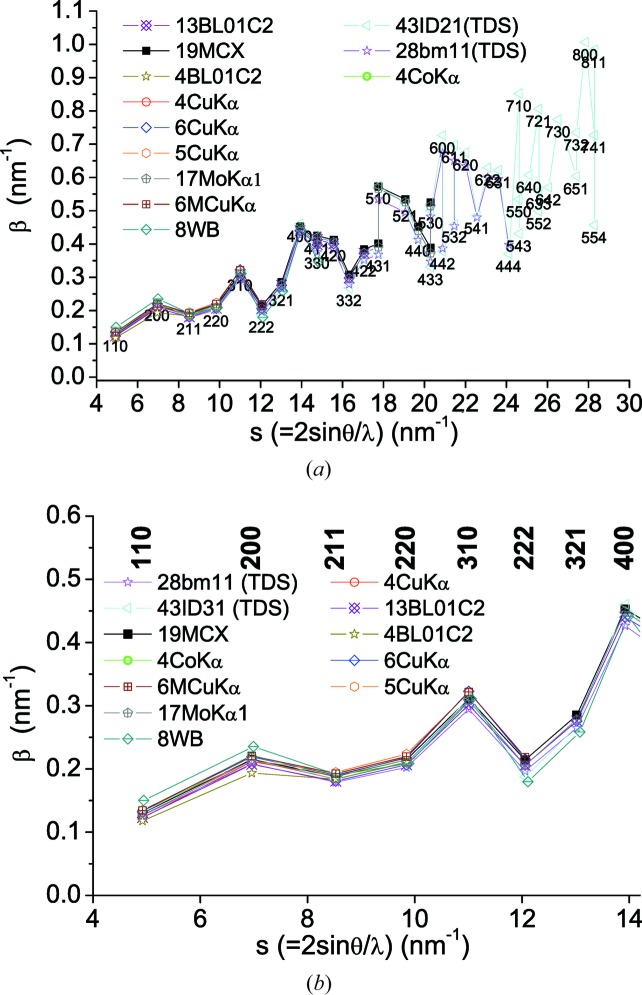
(*a*) Integral breadth, β(*s*), as a function of *s* = 2sinθ/λ, for all data in the present study. A detail of the low-*s* region is shown in (*b*).

Fig. 8 ▸ shows the IB trends for all samples in this study, with a scattering of values which is characteristic of the elastic anisotropy of iron (Rebuffi *et al.*, 2016 ▸).

The trend of IB *versus s* can be modelled by the mWH expression. Differently from the original WH method, which provides for linear dependence of IB on *s*, the mWH equation entails dependence on *s* ^2^ (Ungár *et al.*, 2001 ▸; Scardi *et al.*, 2004 ▸):
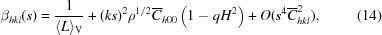
where 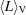 is the volume-weighted mean column length, ρ is the dislocation density and *k* is a parameter related to the dislocation system (interaction/arrangement) (Guinebretière, 2007 ▸; Scardi *et al.*, 2004 ▸). The average contrast factor, 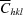, defined in equation (12) for cubic materials, can also be written as
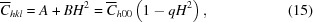
so that 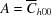 and 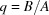 in equation (14). From a practical point of view, equation (14) can be written as
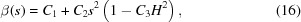
where 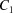, 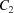 and 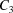 can be refined against experimental data.

**Figure 9:**
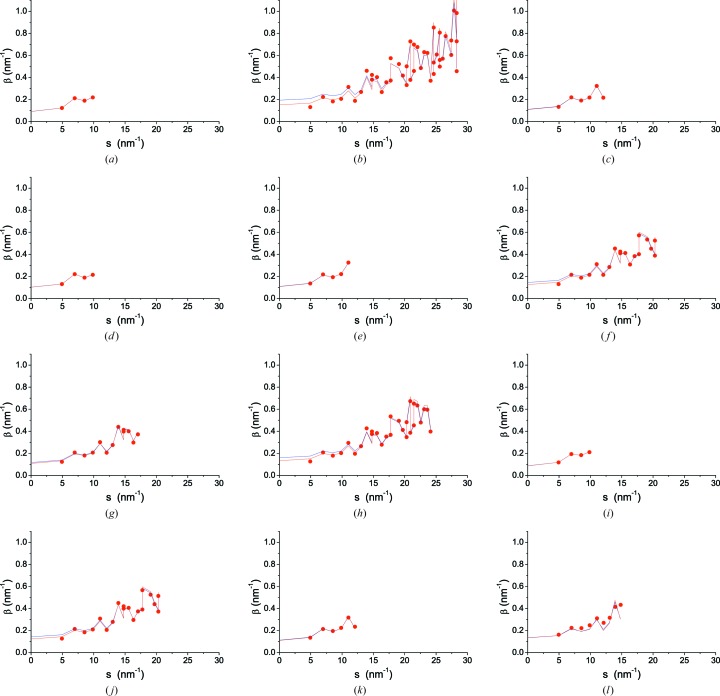
Results of the mWH analysis. Data points (circles) from equation (13); fit by least squares using equation (16) without (blue line) or with (red line) weights (relative integrated intensity). With few data points the two trends, with and without weights, overlap nearly identically. (*a*) 4CuKα; (*b*) 43ID22; (*c*) 6CuKα; (*d*) 4CoKα_1_; (*e*) 5CuKα; (*f*) 19MCX; (*g*) 13BL01C2; (*h*) 28bm11; (*i*) 4BL01C2; (*j*) 17MoKα_1_; (*k*) 6MCuKα; (*l*) 8WB.

The results of fitting equation (16) to the IBs given by equation (13) are shown in Fig. 9 ▸, where blue and red lines stand, respectively, for least-squares fits of equation (16) without and with weights. The weights were the relative integrated intensities of the peaks. The use of weights has no effect when only a few peaks are used, whereas it gives significant (and more credible) results when many peaks are considered. The data in Table 2 ▸ are from weighted fitting.

If we assume, coherently with the assumptions used so far, that crystalline domains are represented by spheres, then 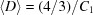. This allows for a direct comparison of domain size results with those provided by WPPM, as reported in Table 2 ▸. Interpretation of 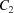 and 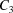 is less straightforward than that of 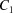. In fact, we cannot directly compare 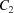 with previous (WPPM) results, other than to point out that, ideally, all data sets should give the same result, whereas the deviations approach 100% (clearly larger than in the Warren plots of Fig. 7 ▸). 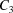 can be compared with the corresponding anisotropy parameter (−*B*/*A*) refined by WPPM, showing reasonable consistency in Table 2 ▸.

Owing to instability (few peaks, noisy data, high background), WPPM of 4BL01C2 was performed while keeping σ^2^ (variance of the lognormal size distribution) fixed. The 17MoKα_1_ data, although providing results not far from those of SR data, tend to instability in the least-squares modelling because of the high background, in particular the sloping trend of the region of the most intense reflections of iron (Fig. 4 ▸). This is probably a consequence of removing the receiving slit, or keeping the antiscatter slit wide open, both allowing high count rates but responsible, at the same time, for the high background signal and high 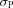 (Fig. 5 ▸).

Note that the agreement between the mWH model and data is better the lower the number of data points. This apparent paradox, caused by using nearly as many parameters as data points, suggests caution in the interpretation of the IB analysis. A good match with few data points should not increase confidence in the reliability of numerical results.

In summary, the anisotropy of microstrain is correctly described by 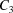, while the 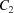 parameter, besides providing a much less definite description of the microstrain effect than is possible by WPPM, varies considerably. 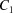 gives just an estimate of the mean domain size, as its values scatter more and are systematically larger than those given by WPPM. This is related to the weakness of the hypotheses underlying all IB methods, including lack of consideration for the size dispersion (distribution variance), arbitrary additivity of size and strain terms, and use of an extrapolation to *s* = 0 to provide information on the mean domain size.

**Figure 10:**
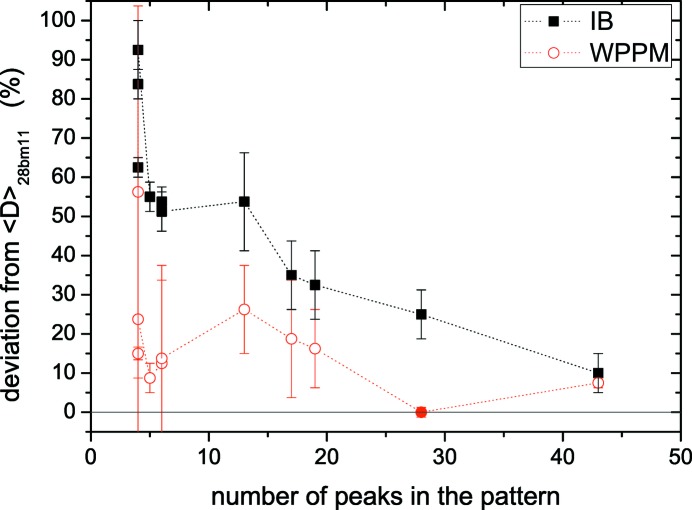
Deviation of mean domain size from the reference WPPM value for 28bm11 data, (〈*D*〉 − 〈*D*〉_28bm11_)/〈*D*〉_28bm11_, as a function of the number of peaks in the pattern. The mean size is calculated by the integral breadth (mWH) method (squares) and by WPPM (open circles; filled circle for the reference data).

The discrepancy between mean size values obtained by mWH and WPPM is not random, but increases as the number of peaks in the pattern decreases (Table 2 ▸). This is better shown in Fig. 10 ▸, where the mean size given by WPPM for 28bm11 is taken as a reference to calculate the percentage deviation of all other results. Systematic deviation (overestimation) of the mean size is large when just a few peaks are used in the IB analysis, whereas WPPM seems much less affected. It is then confirmed that IB methods should be used with caution, especially when few Bragg peaks are available; it would be better to use the results for a qualitative analysis than for a quantitative assessment and separation of size–strain effects, for which a modelling of the whole powder pattern is more reliable and informative.

## 6. Conclusions   

The separation of size and strain effects in line profile analysis was the object of a round robin involving 11 laboratories routinely operating powder diffractometers. The main purpose of the study was to assess the reliability of LPA, and how the result is influenced by the quality and quantity of information available from experiments performed with different optics and X-ray wavelengths, using both commercially available equipment and synchrotron radiation XRD instruments. The sample analysed, a ball milled iron alloy powder, is considered an ideal case as the domain size and strain contributions are of similar magnitude and large enough to separate from the instrumental contribution.

As expected, SR XRD data give the best quality results. In addition to the high count rate, the large number of peaks in the pattern typical for high-energy beamlines is key in improving the separation of size and strain with respect to the stability and reliability of the refinements used. Furthermore, the ability to measure XRD patterns at different temperatures allows for consideration of the diffuse scattering, provided that intensities are accurately measured. This condition can be achieved using Debye–Scherrer geometry and high-energy beams, making capillary absorption negligible. Nevertheless, LPA can provide acceptable results even with data collected by easily accessible laboratory instruments. This is also true when low-energy radiation is used, which allows a limited number of peaks to be observed, as long as the statistical quality is good.

The round robin results show that, for the best results, data should be collected with (i) high counts and (ii) low smooth background, both contributing to good counting statistics, *i.e.* to a low standard deviation of the intensity distribution. Stable results and reliable size–strain separation are supported by (iii) high-energy X-ray radiation, to collect as many Bragg peaks as possible, but also require (iv) proper consideration of the IP, determined under identical experimental conditions *via* the available standard powder XRD samples.

Although the study did not provide a comprehensive comparison of the different LPA methods, the superiority of the methods based on the analysis of the entire profile is clear compared to those that use only the width of the diffraction peak. This difference is particularly evident when few peaks are present in the experimental pattern; IB methods, like the modified Williamson–Hall plot, can provide a perfect match to the limited experimental data but clearly overestimate the domain size, whereas Fourier methods, like the WPPM used in this study, limit the overestimation to within acceptable limits.

## Supplementary Material

Supporting information file. DOI: 10.1107/S1600576718005411/ks5592sup1.pdf

## Figures and Tables

**Table 1:** List of instruments used in this study and essential information on measurement conditions Details are reported in the supporting information. Energy; measurement geometry, optics and detector (scint. for scintillator; mon. for monochromator); line profile standard (NIST Standard Reference Materials; Cline *et al.*, 2000 ▸, 2010 ▸); number of peaks; sampling step; total counting time in the measurement.

Name	Energy (keV)	Geometry and detector	Line profile standard	Number of peaks	Step (°)	Total counting time (s)	Time per step (s)
Synchrotron radiation sources							
43ID22	31	Debye–Scherrer	SRM660b	43	0.01	4200	0.667
		9 scint./(111)Si mon.					
28bm11	30	Debye–Scherrer	SRM660b	28	0.005	2760	0.3
		12 scint./(111)Si mon.					
13BL01C2	24	Imaging plate	SRM660a	13	0.0196	360	
4BL01C2	18	Imaging plate	CeO_2_	4	0.0168	30	
19MCX	15	Debye–Scherrer	SRM660a	19	0.05	61230	30
		1 scint./(111)Si mon.					
Laboratory sources							
17MoKα_1_	17	Transmission/capillary	SRM660a	17	0.0114	50407	7 × 192
		PSD (192 channels)					
6MCuKα	8	θ/2θ-Goebel mirror	SRM660a	5–6	0.0307	39100	10 × 192
		PSD (192 channels)					
6CuKα	8	Bragg–Brentano	SRM660	6	0.1	70920	60
		1 scint./graphite mon.					
4CuKα	8	θ/θ	SRM660	4	0.03	14634	6
		1 scint./graphite mon.					
5CuKα	8	Bragg–Brentano	SRM660b	5	0.0755	9540	6 × 192
		PSD (192 channels)					
4CoKα_1_	7	Bragg–Brentano	SRM660b	4	0.0105	231624	18 × 192
		PSD (192 channels)					
8WB†	0.1–26.2	Energy dispersive	SRM660a	8	[Table-fn tfn1]	2800	400
		Si-drift detector					

† Different sampling steps for the data collected at seven angular positions of the Si drift detector: see supporting information for details.

‡ For details see Mendoza Cuevas *et al.* (2015 ▸).

**Table 2:** Comparison of the results of mWH analysis [equation (16)] with mean domain size from the WPPM of Figs. 4, 6 and 7, and parameter 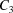 with *B*/*A* from WPPM [*cf*. equations (12) and (15)]

	Mean domain size 〈*D*〉 (nm)					
Pattern	mWH	WPPM	*C*_2_	*C*_3_	WPPM *B*/*A*	
28bm11	10.0 (0.5)	8.0 (0.1)	0.0013	1.87	2.12	
43ID22	8.8 (0.4)	8.6 (0.1)	0.0013	2.02	2.33	
13BL01C2	12.3 (1.0)	10.1 (0.9)	0.0018	1.80	2.01	
4BL01C2†	15.4 (0.6)	9.9 (3.3)	0.0022	1.62	1.60	
19MCX	10.6 (0.7)	9.3 (0.8)	0.0016	1.81	2.01	
4CoKα_1_	13.0 (0.2)	9.2 (0.13)	0.0024	2.07	2.11	
6CuKα	12.3 (0.3)	9.0 (2.0)	0.0022	1.98	2.03	
4CuKα	14.7 (0.3)	12.5 (3.8)	0.0025	1.86	1.87	
5CuKα	12.4 (0.3)	8.7 (0.3)	0.0021	1.76	1.95	
17MoKα_1_	10.8 (0.7)	9.5 (1.2)	0.0016	1.88	2.08	
6MCuKα	12.1 (0.4)	9.1(1.6)	0.0022	1.96	1.79	
8WB	9.9 (2.0)	10.4(5.0)	0.0018	2.23	1.41	

† σ^2^ in equation (9) (variance of lognormal distribution) was fixed during WPPM owing to instability of the fit with just four peaks on a relatively high background.
