# Supplementary material for: Size–strain separation in diffraction line profile analysis
Source: J Appl Crystallogr. 2018 May 29;51(Pt 3):831–43. doi: 10.1107/S1600576718005411 (PMC5988009; doi:10.1107/S1600576718005411)
Supplement: Supplementary file 1 [file j-51-00831-sup1.pdf]

JOURNAL OF APPLIED CRYSTALLOGRAPHY

Volume 51 (2018)

Supporting information for article:

Size-strain separation in diffraction Line Profile Analysis

P. Scardi, M. Ermrich, A. Fitch, E-Wen Huang, R. Jardin, R. Kuzel, A. Leineweber, A. Mendoza Cuevas, S. T. Misture, L. Rebuffi and Christian Schimpf

P. Scardi[1,*], M. Ermrich[2], A. Fitch[3], E-Wen Huang[4], R. Jardin[5], R. Kuzel[6], A. Leineweber[7], A. Mendoza Cuevas[8,9], S.T. Misture[10], L. Rebuffi[11], C. Schimpf[7]

[1] Department of Civil, Environmental & Mechanical Engineering, University of Trento, Trento, Italy

[2] Röntgenlabor Dr. Ermrich, Am Kandelborn 7, D - 64354 Reinheim, Germany

[3] ESRF, 71, avenue des Martyrs, CS 40220, 38043 Grenoble Cedex, France

[4] Bruker AXS GmbH, Oestliche Rheinbrueckenstrasse 49, 76187 Karlsruhe, Germany

[5] Department of Materials Science & Engineering, National Chiao Tung University, Hsinchu, Taiwan

[6] Department of Condensed Matter Physics, Charles University, Prague, Czech Republic

[7] Institute of Materials Science, TU Bergakademie Freiberg, D-09599 Freiberg, Germany

[8] Archaeometry Laboratory, Havana's Historian Office, University San Geronimo de La Habana, Habana Vieja, Cuba

[9] Multidiscipilanary Laboratory, ICTP, I-34151, Trieste, Italy

[10] Alfred University, Alfred, NY 14802, USA

[11] Elettra-Sincrotrone Trieste, Trieste, Italy

* corresponding author: Paolo.Scardi@unitn.it

S1. Supporting Information

Participating laboratories were not given prescriptions on measurement geometry or representation of the data, but only the recommendation to use a suitable line profile standard, which in most cases was NIST SRM 660 (Cline et al., 2000; 2010). This approach was intentional, to highlight differences in real experimental practice, to understand how different data, collected (i) on different instruments and (ii) in different data collection conditions, perform in a Line Profile Analysis. As a consequence, information on data collection reported in this Supporting Information file is not uniform.

In the following, additional data are presented on the Instrumental Profile and on the statistical quality of data and modelling. As already pointed out in the main text, statistical quality of the data can be assessed from the results of the Whole Powder Pattern Modelling (WPPM), although the different datasets were collected and represented in different forms, a condition which limits the possibility of quantitative comparisons. Some of the datasets were provided as intensity in counts vs 2θ, in a rather standard form, whereas datasets 6CuKα, 5CuKα, 6MCuKα and 17MoKα$_1$ were given in counts per second (cps), which required a multiplication by counting time per step to be compared with the other datasets. 8WB is definitely different as it is collected in energy dispersive mode, which adopts a geometry not specifically optimized for studying the line profile; as a consequence the corresponding σ_P (Figure S1) is expected to be quite large.

Data quality and statistical information

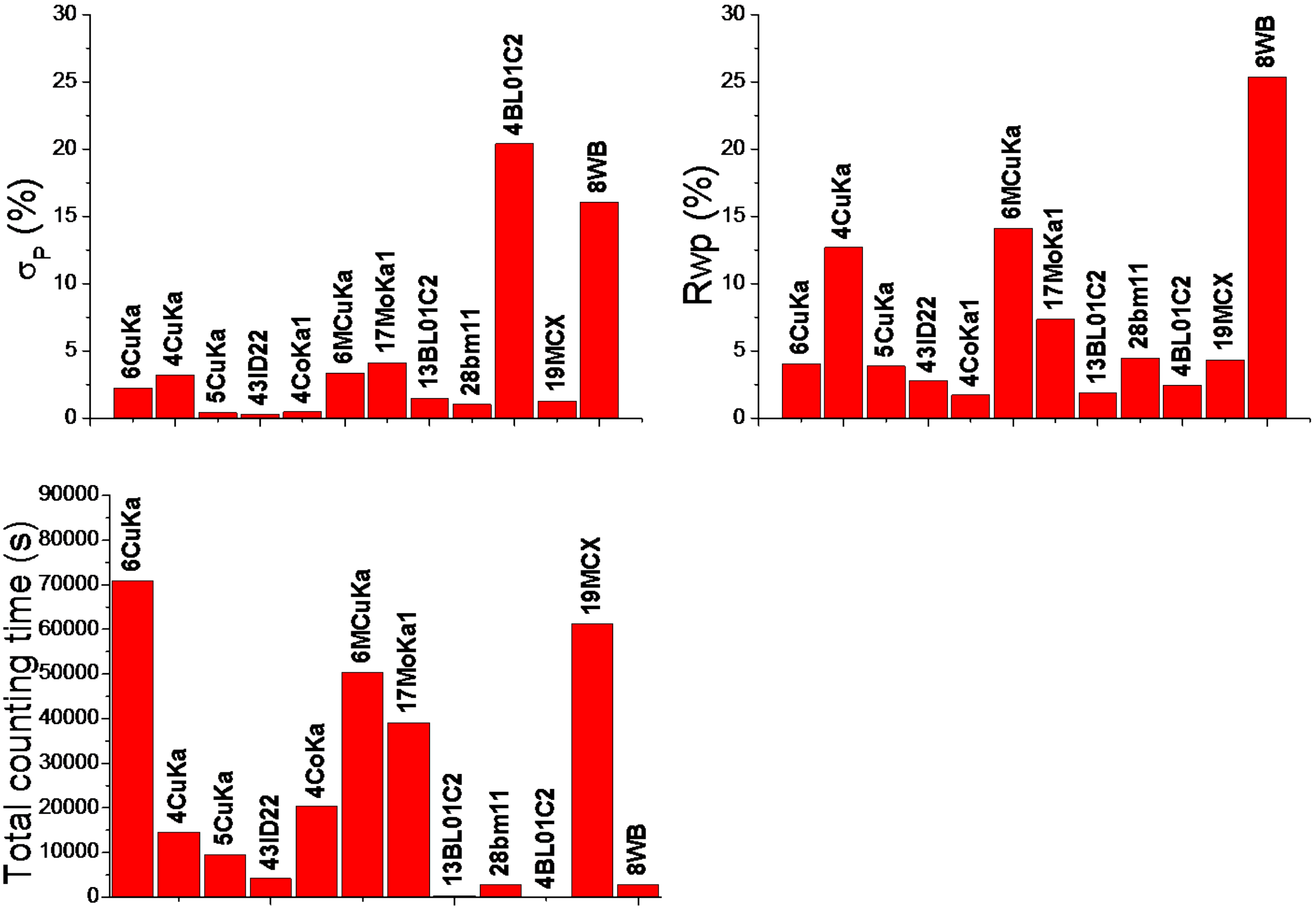

Figure S1. Comparison of counting statistics of the different datasets, expressed as standard deviation of the intensity distribution (Klug & Alexander, 1974), $\sigma_P = \sqrt{N_T + N_B} / (N_T - N_B)$, where N_T is the total intensity (diffraction and

background) and N_B is the background intensity (a); WPPM statistical index, $R_{wp} = \left(\sum_i S_i \Big/ \sum_i w_i y_i^2\right)^{1/2}$, R-weighted pattern (b); total counting time for each dataset collection (from Table I in the main article) (c).

Experimental conditions of data collection

Information is different for each laboratory / instrument used. As pointed out above, this choice was made with the intent to test and compare datasets obtained in a variety of conditions, representative of daily practice of the laboratories involved in this study.

6CuKα

Instrument: XRD-7 Seifert-Freiberg Prazisionmechanik, vertical θ/2θ diffractometer

Tube: Cu

2θ step: 0.1°

Counting time: 60s

Sample holder: flat sample holder, front loaded

Monochromator: graphite, in diffracted beam

Detector: Scintillation counter

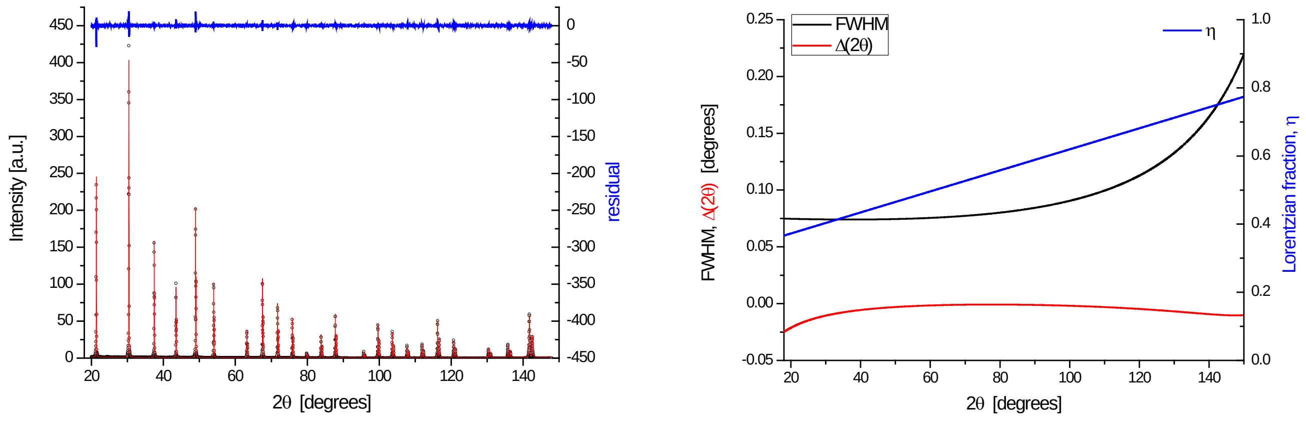

Figure S2. Line profile standard powder pattern (NIST SRM660a): data (circle) and fit (red line) and difference (residual, blue line above) (left); corresponding parameterization of Instrumental Profile and correction for aberrations on Bragg peak positions: see main text for details (right). Data refer to the instrument used to collect *6CuKα* data

4CuKα

Instrument: STOE θ/θ- Diffractometer, Reflection mode

Tube: Cu, U = 40kV, I = 40mA

2θ step: 0.03°

Counting time : 6s

Sample holder: flat sample holder (reflection), front loaded, rotating

Monochromator: graphite (002) plane, in diffracted beam

Detector: Scintillation counter

Slits: 0.75mm, 0.35mm, vertical 2 x 8mm

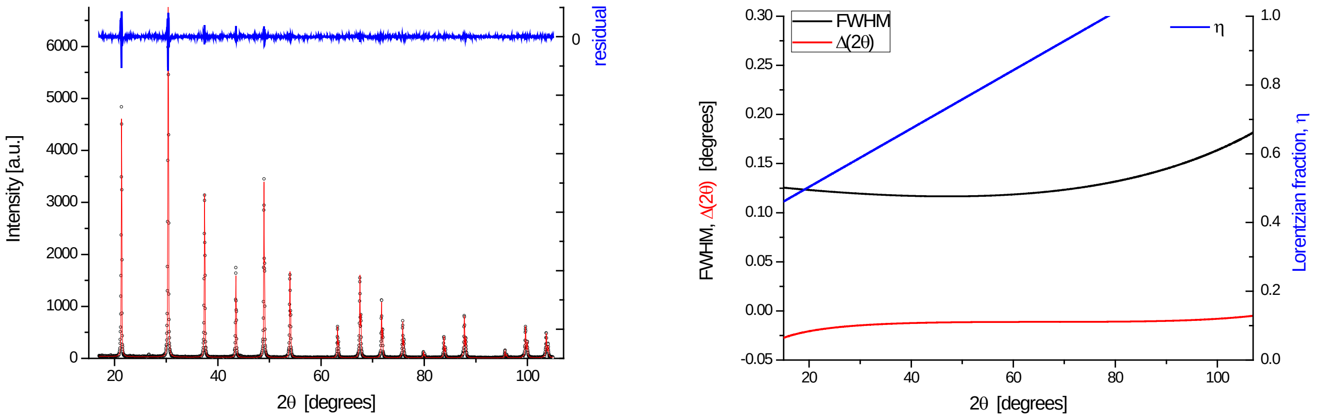

Figure S3. As in Figure S2, instrument used to collect *4CuKα* data

5CuKα

Instrument: Bruker, measurement circle: 500 mm

Tube: Cu Long Line Focus

2θ step: 0.0755°

Counting time: 1152s per step

Sample holder: Si with cavity

Detector: PSD – LynxEye XE-T (3.3° opening)

Slits: divergence slit: 0.3°; axial (Soller) slit, both primary and secondary beam: 2.5°.

Scan from 15 to 135°, actually requires the PSD starts ½ detector opening before (about 13.35°) and finishes ½ detector opening after (about 136.65°). In this way the detector measures each 2θ position in the 15-135° range. The detector opening is 3.3° (192 channels) for a goniometer radius of 250mm. The measurement is done in continuous mode.

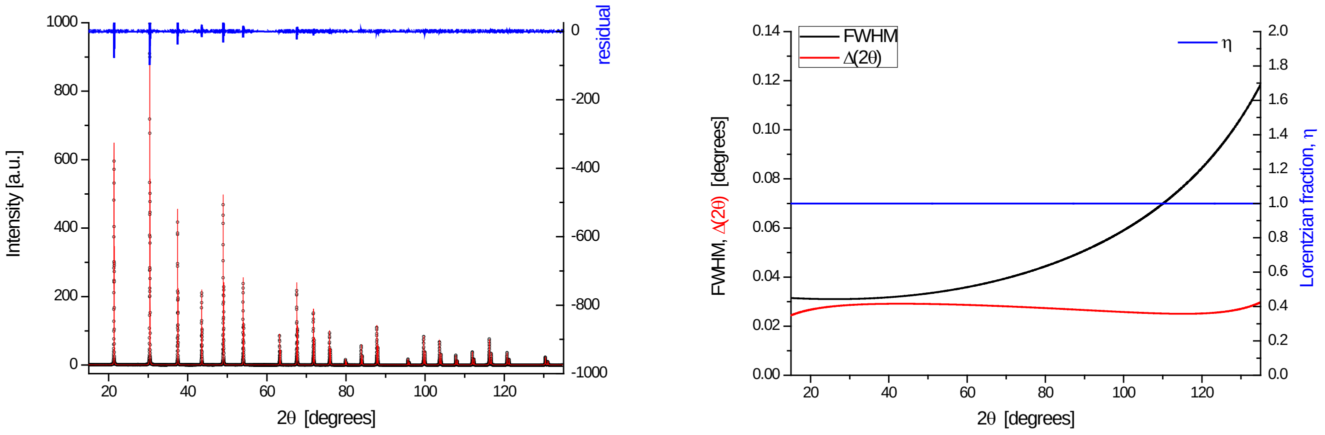

Figure S4. As in Figure S2, instrument used to collect *5CuKα* data

43ID22

Instrument: beamline ID22, ESRF, Grenoble (F)

Energy: 31keV

2θ step: 0.01°, Counting time: 2/3 s (total of 4200 s)

Sample holder: glass capillary 0.3mm

Measurements are made in standard continuous-scanning mode. When merging original data from nine counters, the original 0.0005° step is rebinned to 0.01° of the dataset analysed in this study. Esds on data points are calculated by propagating errors due to summing a number of repeated scans, and the nine counters.

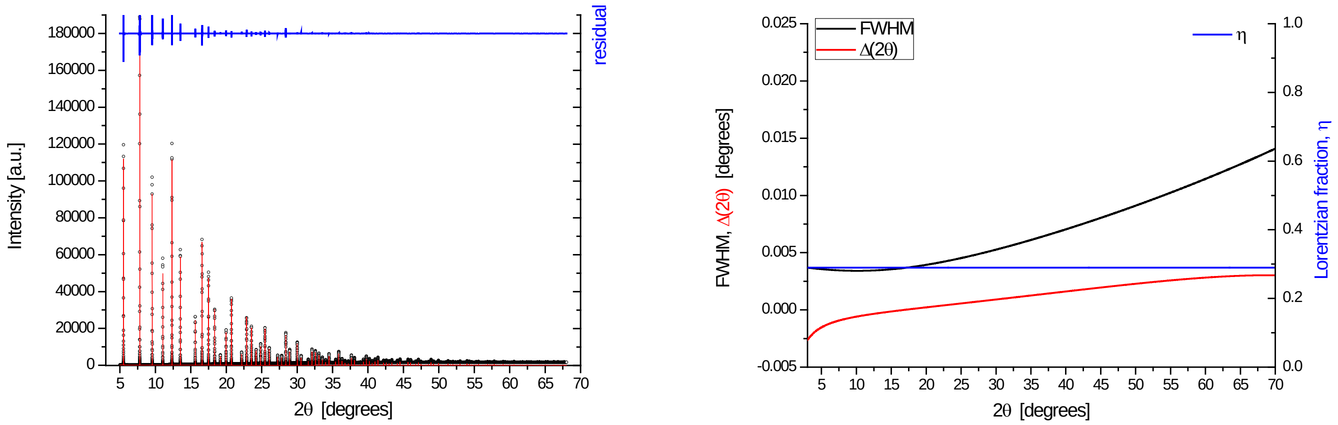

Figure S5. As in Figure S2, instrument used to collect *43ID22* data

4CoKα_1

Instrument: Bruker D8 Advance diffractometer in Bragg-Brentano-geometry, measurement circle 500 mm

Tube: Co anode, operated at 35 kV/35 mA, long-fine focus tube

2θ step: 0.0105° (number of steps: 12868)

Counting time: 9 repetitions with 2 seconds per step in continuous mode (18s), sample spinning

Sample holder: small amount (about a knife tip) of powder dispersed in isopropanole and sedimented as a thin layer on a <510> cut Si substrate

Monochromator: primary beam monochromator; Johannsson focussing geometry (SiO2), Co-Kα1 radiation; focus

Slits: fixed divergence slit, 0.6 mm; secondary beam, 2.5° vertical Soller slit; monochromator focus slit, 0.1 mm

Detector: PSD – Lynxeye

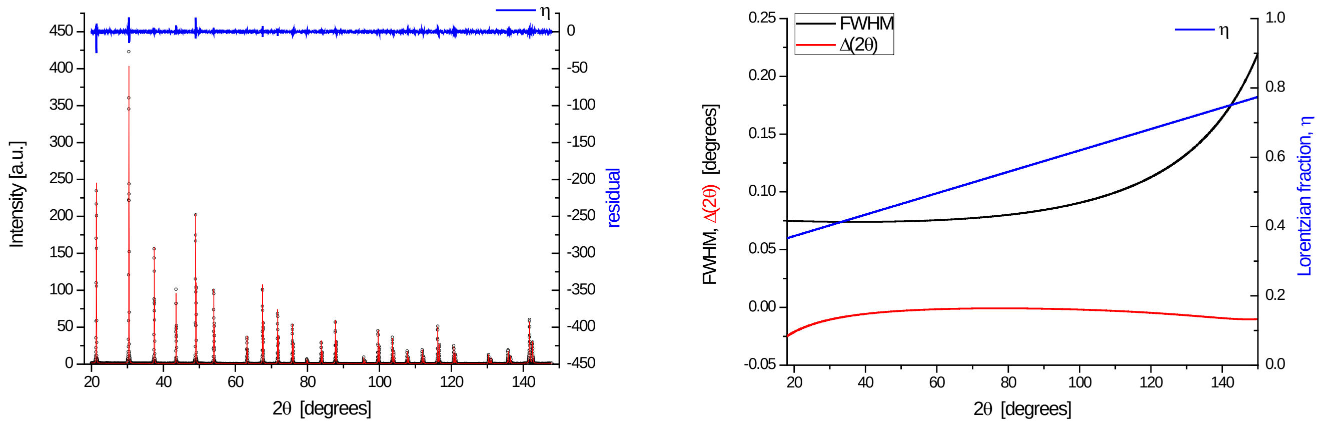

Figure S6. As in Figure S2, instrument used to collect *4CoKα_1* data

6MCuKα

Instrument: Bruker, Gobel Mirror (0.2 mm primary beam aperture), coupled 2Θ/Θ scan without knife edge

Tube: Cu anode

Number of steps: 3910

Counting time: total time per step, 1920 s

Detector: PSD –Lynxeye; energy discriminator was set to VLL = 0.216 V and VUL = 0.256 V.

Slits: fixed divergence slit: 0.5°, axial divergence (Soller) slits, 2.5° on primary beam side and on detector side.

Detector antiscatter slit was fully open to 6.974°, but PSD detector window was set to 2.947° (measurement with variable divergence slit: sample length was fixed to 15 mm (primary and detector slit)).

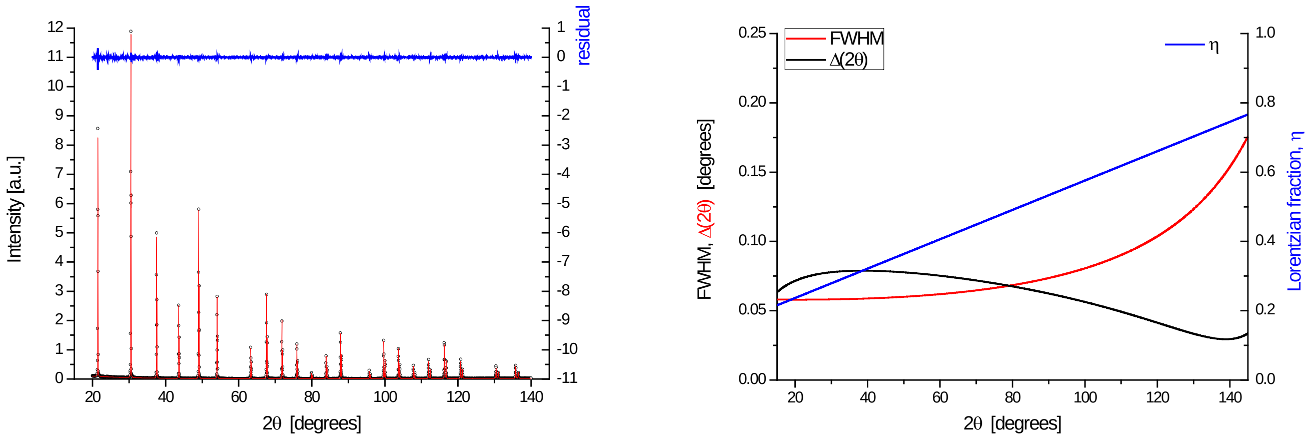

Figure S7. As in Figure S2, instrument used to collect *6MCuKα* data

17MoKα1

Instrument: Bruker; crystal focus is on the detector.

Energy: 17 keV / Wavelength nm.

Number of steps: 7201

Counting time: total time per step, 1344 s

Sample holder: 0.3mm capillary

Detector: PSD, Bruker Lynxeye detector, with thick crystal to improve efficiency at Mo radiation

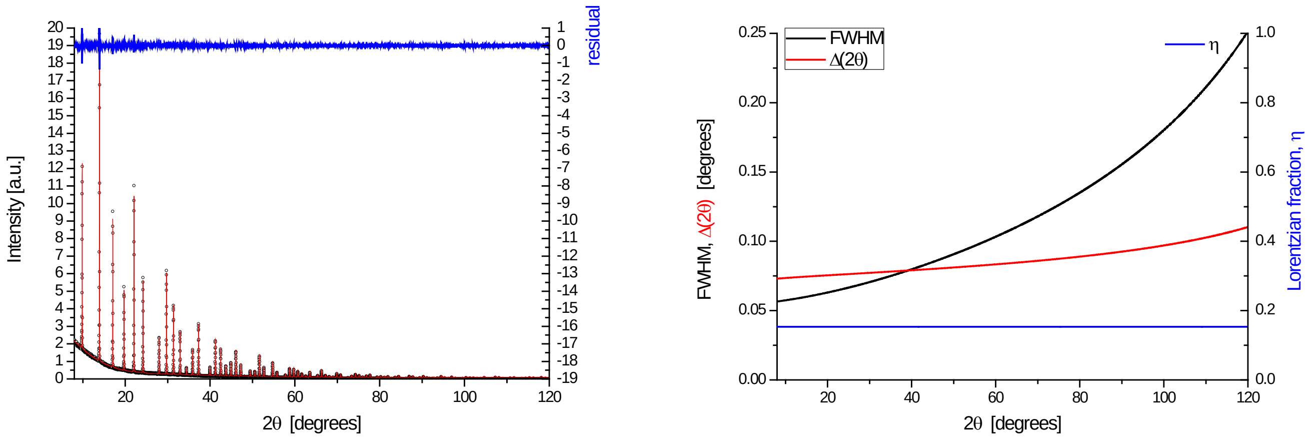

Figure S8. As in Figure S2, instrument used to collect 17*MoKα₁* data

13BL01C2

Instrument: beamline BL01C2, NSRRC, Hsinchu, Taiwan

Energy: 24 keV / Wavelength_0.051nm

Data Collection time: 360s

Sample holder: glass capillary 0.2mm, wall thickness 0.01mm

Detector: 2D MAR

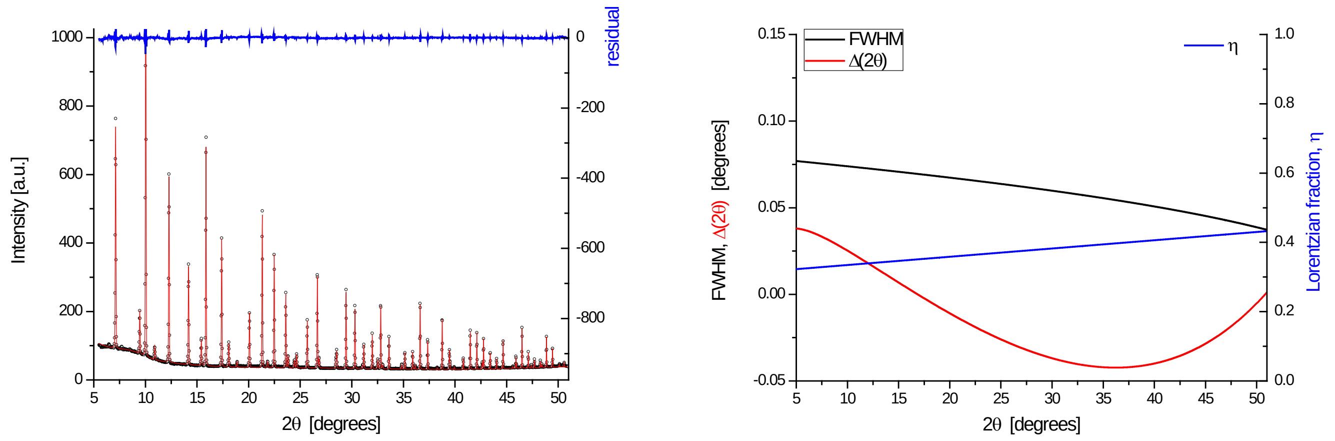

Figure S9. As in Figure S2, instrument used to collect *13BL01C2* data. In this case the standard powder was a mixture of NIST SRMs, LaB_6 and CeO_2; only the former one was used to determine the Instrumental Profile.

28bm11

Instrument: 11-BM, the powder diffraction beamline at the Advanced Photon Source (Argonne National Laboratory, Illinois, USA).

Energy: 30 keV / CalibratedWavelength_0.0413679nm

2θ step: 0.005°

Counting time: 0.3s

Sample holder: kapton capillary (radius R = 0.15 mm); powder was diluted in carbon black: $\mu R < 0.1$ at 30 keV, sufficiently small to make absorption corrections unnecessary.

XRPD data on the same sealed capillary were collected at 100, 200 and 300 K, in sequence, using an air blower to condition the capillary temperature

Detector: 12 scintillators, each one with (111) Si monochromator

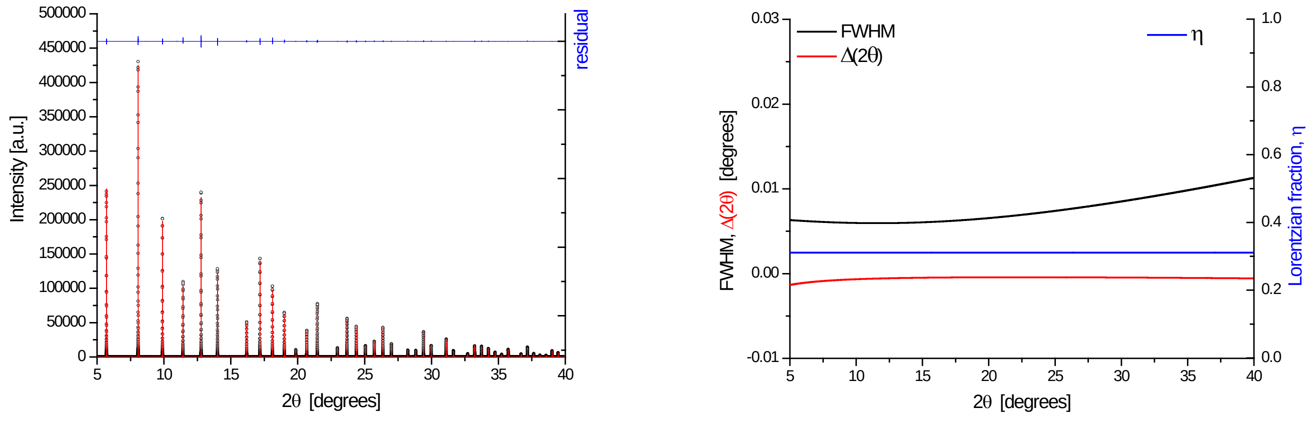

Figure S10. As in Figure S2, instrument used to collect *28bm11* data

4BL01C2

Instrument: beamline BL01C2, NSRRC, Hsinchu, Taiwan

Energy: 18 keV / CalibratedWavelength_0.069nm

Data Collection time: 30s

Sample holder: glass capillary 0.2mm

Detector: 2D

The pattern was collected as a test dataset, reason why data collection time was so short. Instrumental profile was determined in the following data collection turn (here labelled as 13BL01C2), and used also for the present set of 4BL01C2 data.

19MCX

Instrument: beamline MCX, Elettra Sincrotrone, Trieste, Italy

Energy: 15 keV (λ= 0.8265 nm).

2θ step: 0:05°

Counting time: 30 s (special counting time for the long-2θ range measurement of batch 4A)

Monochromator: (111) Silicon in diffracted beam

Sample holder: kapton capillary (diameter XX μm), spun at 3000 rpm.

Detector: 1 scintillator

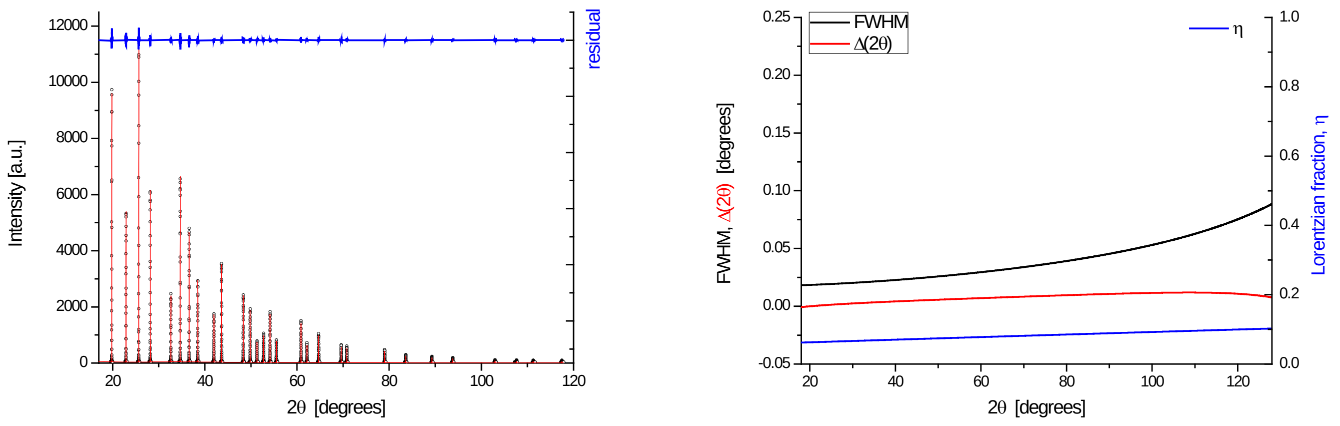

Figure S12. As in Figure S2, instrument used to collect *19MCX* data

8WD

Instrument: multiple-angle energy dispersive XRD (EDXRD) system. A portable instrument based on a low-power miniaturized X-ray tube and Si-drift detector mounted on a motorized stage allowing angular scansion. Details can be found in Mendoza Cuevas *et al.* (2015). The powder pattern of the ball milled Fe-1.5wt%Mo specimen was obtained by merging data collected at 7 different angular positions of the Si-drift detector.

Tube: Ag anode

Counting time: 400s per observed angle (7 angles for the ball milled Fe-1.5wt%Mo powder)

Sampling step: in q space, from 0.0039 $Å^{-1}$ for (110) to 0.00734 $Å^{-1}$ for (330)/(411) peaks of the studied powder

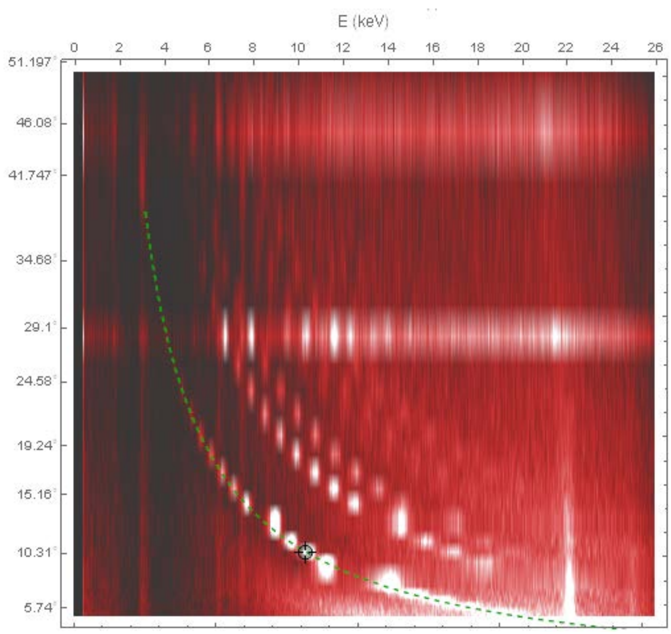

(a)

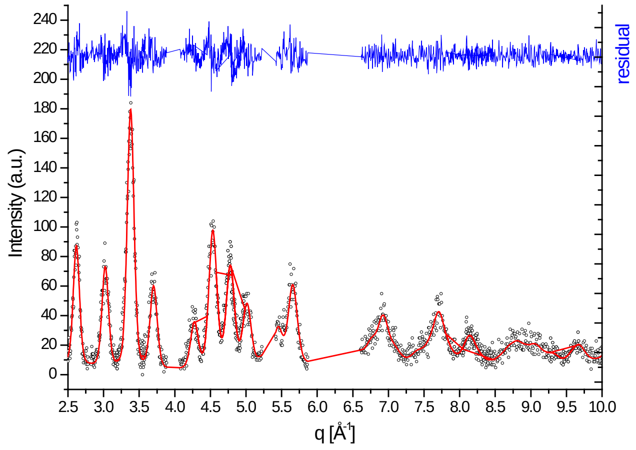

(b)

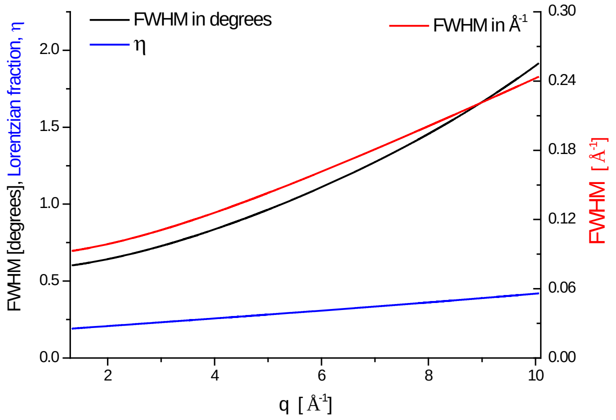

(c)

Figure S13. Same standard powder as in Figure S2 (NIST SRM660a) measured in energy dispersive data collection mode. Example of density plot, angle vs. energy (E) in keV (a); SRM660a powder pattern in q scale (=2πs = 4πEsin(θ)/12398, where θ is calculated for Bragg peaks of standard LaB_6, unit cell parameter 4.1569162 Å (Cline *et al.*, 2000)): data (circle), modelling (red line) and difference (residual, blue line above) (b); parameterization of the Instrumental Profile component (see main text for details) (c): in addition to the FWHM in q space (right ordinate axis), we also show the FWHM in degrees (left ordinate axis), calculated for a wavelength λ=0.7093 Å (MoKα1), so to allow an easier comparison of IP, e.g., with that shown in Figure S8.

References

Cline, J.P., Deslattes, R. D., Staudenmann, J-L. , Hudson, L.T., Henins, A. & Cheary, R.W. (2000). Certificate SRM 660a. NIST, Gaithersburg, Maryland, USA.

Cline, J.P., Black, D., Windover, D. & Henins, A. (2010). Certificate SRM 660b. NIST, Gaithersburg, Maryland, USA.

Klug, H.P. & Alexander, L.E. (1974). *X-ray Diffraction Procedures*, 2nd ed. New York: John Wiley.

Mendoza Cuevas, A., Bernardini, F., Gianoncelli, A., and Tuniz, C. (2015). X-ray Spectrometry **44** [3], 105–115. doi: 10.1002/xrs.2585.
